# Early Retirement: A Meta-Analysis of Its Antecedent and Subsequent Correlates

**DOI:** 10.3389/fpsyg.2017.02157

**Published:** 2018-01-04

**Authors:** Gabriela Topa, Marco Depolo, Carlos-Maria Alcover

**Affiliations:** ^1^Social and Organizational Psychology, Universidad Nacional de Educación a Distancia, Madrid, Spain; ^2^Psychology, Università di Bologna, Bologna, Italy; ^3^Medicine and Surgery, Psychology, Preventive Medicine and Public Health, Immunology and Medical Microbiology, Nursing, and Stomatology, Universidad Rey Juan Carlos, Madrid, Spain

**Keywords:** retirement, early retirement, meta-analysis, voluntary retirement, aging

## Abstract

Early or voluntary retirement (ER) can be defined as the full exit from an organizational job or career path of long duration, decided by individuals of a certain age at the mid or late career before mandatory retirement age, with the aim of reducing their attachment to work and closing a process of gradual psychological disengagement from working life. Given the swinging movements that characterize employment policies, the potential effects of ER—both for individuals and society—are still controversial. This meta-analysis examined the relationships between ER and its antecedent and subsequent correlates. Our review of the literature was generated with 151 empirical studies, containing a total number of 706,937 participants, with a wide range of sample sizes (from *N* = 27 to *N* = 127,384 participants) and 380 independent effect sizes (ESs), which included 171 independent samples. A negligible ES value for antecedent correlates of early retirement (family pull, job stress, job satisfaction, and income) was obtained (which ranged from *r* = −0.13 to 0.19), while a fair ES was obtained for workplace timing for retirement, organizational pressures, financial security, and poor physical and mental health, (ranging from *r* = 0.28 to 0.25). Regarding ER subsequent correlates, poor ESs were obtained, ranging from *r* = 0.08 to 0.18 for the relationships with subsequent correlates, and fair ESs only for social engagement (*r* = −0.25). Examination of the potential moderator variables has been conducted. Only a reduced percentage of variability of primary studies has been explained by moderators. Although potential moderator factors were examined, there are several unknown or not measurable factors which contribute to ER and about which there are very little data available. The discussion is aimed to offer theoretical and empirical implications suggestion in order to improve employee's well-being.

## Introduction

Most developed and developing countries are experiencing demographic changes characterized both by decrease in fertility and increase in longevity. Population aging is a global phenomenon in Europe, North America, and the Asia-Pacific Region, and is often accompanied by relatively low employment rates for people aged 55 – 64 years, ranging from 73.6% in Japan, 65.0% in G7 countries, 64.1% in USA, and 59.1% in EU 28 to 53.4% in Italy and, for people 65 years and over, ranging from 31.5% in Korea, 19.3% in USA, 14.9% in G7 countries, and 5.7% in EU 28 to 2.0 in Spain (OECD, 2017, data from 2016). The context of high unemployment, industrial restructuring, and negative stereotypes of aged workers have contributed, especially in the last two decades of the twentieth century, to the institutionalization of early retirement (hereafter, ER) as a specific employment policy to make room for younger employees (Von Nordheim, 2004; Kim, 2009; Engelhardt, 2012). However, empirical data do not support the hypothesis that employment of the young and old are interchangeable, and suggest that encouraging later retirement will have no adverse effect on youth employment (Kalwij et al., 2010). Moreover, the welfare states have financially encouraged early withdrawal before age 60, and ER has been considered an individual right or a desirable new social status that guarantees high incomes. For decades, ER has been popular in European countries and was seen as a much appreciated social claim that increases personal satisfaction and well-being, particularly among workers who perceive poor health or who suffer from work-related health problems (Börsch-Supan and Schuth, 2014).

As a consequence, ER affected millions of workers by the end of the year 2000. With the arrival of the financial crisis and the economic downturn in 2008, the pension reform debate gained new force, and developed countries increased their pressure to reduce ER schemes, along with policies to improve the employability of aged workers. The generalized shift from “pro-retirement” to “pro-work” (Wang and Shultz, 2010) detected in the last decade was driven by a confluence of the needs of governments, employers, and of the older persons themselves (Komp et al., 2010). Increasing the statutory retirement age to stimulate older people to continue working, make ER financially unattractive, and make greater efforts to retain older workers in organizations are unavoidable challenges both for governments and firms (Truxillo and Fraccaroli, 2013; Bal et al., 2015). Given these swinging movements that characterize employment policies, the potential effects of ER—both for individuals and society—are still controversial (Feldman, 2003, 2013; Henkens and van Dalen, 2003; Cleveland and Lim, 2007).

## Defining ER

ER can be defined as the full exit from an organizational job or career path of long duration, decided by individuals of a certain age at the mid or late career before mandatory retirement age, with the aim of reducing their attachment to work and closing a process of gradual psychological disengagement from working life (Beehr, 1986; Feldman, 1994; Schalk and Desmette, 2015). Previous research defined ER according to three objective criteria: age, years of service, and eligibility. But, as recent reviews of literature stated, “these objective criteria still play a major role in understanding ER decisions, but exclusive reliance on these criteria may no longer be warranted” (Feldman, 2013, p. 281). According to this recommendation, it seems necessary to take into account subjective definitions of ER, typically emerging as older workers' perceptions and attitudes about whether it is time to retire. First, normative age for retirement is the result of comparison with others in the same occupation or organization. Second, individuals view themselves as retirees, especially if they have maintained psychological distance from their work. Third, later career and life stages trigger identification with non-work roles, which in turn should drive ER decisions. Summing up, the social significance of ER has changed in the last decades and, as a consequence, both objective and subjective definitions of ER must be taken into account (Feldman, 2013; Fisher et al., 2016).

Since the pioneer works highlighted the more relevant factors of ER (Beehr, 1986; Hanisch and Hulin, 1991; Ekerdt and De Viney, 1993; Feldman, 1994; Taylor and Shore, 1995; Shultz et al., 1998; Szinovacz, 2003), a great amount of empirical studies has been published (Keith, 1985; Monahan, 1985; Hayward, 1986; Bell et al., 1989; Knesek, 1989; Swan et al., 1991; Härkäpää, 1992; Hardy and Quadagno, 1995; Cunningham, 1996; Eastman, 1996; Gowan, 1998; Edén et al., 1999; Hardy and Hazelrigg, 1999; Kerkhofs et al., 1999; Börsch-Supan, 2000; Kim and Feldman, 2000; Suh, 2000; Bahman, 2001; Rojanawon, 2001; Kim and Moen, 2002; Austrom et al., 2003; Davis, 2003; Elovainio et al., 2003; Kim, 2003; Martínez et al., 2003; Mein et al., 2003; Pauwels, 2003; Szinovacz, 2003, 2013; Tunceli, 2003; Cardano et al., 2004; Husemoen et al., 2004; Karpansalo et al., 2004; Simbula et al., 2004; Szinovacz and Davey, 2004; Blekesaune and Solem, 2005; Buxton et al., 2005; De Judicibus and McCabe, 2005; Hansez et al., 2005; Kiessling and Henriksson, 2005; Rasmussen and Andersen, 2005; Seitsamo, 2005; Taskila-Åbrandt et al., 2005; Tsai et al., 2005; Tuohy et al., 2005; Butterworth et al., 2006; Enthoven et al., 2006; Graves, 2006; Hartig and Fransson, 2006; Rennemark and Berggren, 2006; Vaillant et al., 2006; Bronchetti, 2007; Desmarez et al., 2007; Harkonmäki, 2007; Harkonmäki et al., 2007, 2009; Schuring et al., 2007; Tian, 2007; Aspnes, 2008; Boumans et al., 2008; Carlsen et al., 2008; Desmette and Gaillard, 2008; Schils, 2008; Schnabel et al., 2008; Sharma et al., 2008; Taylor et al., 2008; Brougham and Walsh, 2009; Herrbach et al., 2009; Ilchuk, 2009; Johnston and Lee, 2009; Warren, 2009; Westerlund et al., 2009; Euwals et al., 2010; Pfleger et al., 2010; Pit et al., 2010; Roberts et al., 2010; Shim and Amick, 2010; von Bonsdorff et al., 2010a; Chang and Yen, 2011; Damkjæ et al., 2011; Damman et al., 2011, 2015; Garcia, 2011; Green, 2011; Korkeila et al., 2011; Markkula et al., 2011; Sargent-Cox et al., 2011; Schneider et al., 2011; Schofield et al., 2011, 2013; Smith et al., 2011; Uggerby et al., 2011; Wuebbeke, 2011; Griffin et al., 2012; Helvik et al., 2012; Lahelma et al., 2012; Olesen et al., 2012; Paradise et al., 2012; Böttcher et al., 2013a,b; Neuner et al., 2013; Newman et al., 2013; Ranzi et al., 2013; Segel-Karpas et al., 2013; Wedegaertner et al., 2013; Dewa et al., 2014; De Wind et al., 2014; Horner, 2014; Kuhlman et al., 2014; Laires and Gouveia, 2014; Lindbohm et al., 2014; Mosca and Barrett, 2014; Osler et al., 2014; Singer et al., 2014; Van Droogenbroeck and Spruyt, 2014; Dal Bianco et al., 2015; De Wind and van der Beek, 2015; Janković et al., 2015; Lawless et al., 2015; Morois et al., 2015; Plouvier et al., 2015; Robroek et al., 2015; Ahomäki et al., 2016; Carr et al., 2016; Frilander et al., 2016; Hagger-Johnson et al., 2016; Ihle et al., 2016; Jensen et al., 2016; Laires et al., 2016; Myhr et al., 2016; Nilsson et al., 2016; Thorsen et al., 2016; Whitney et al., 2016). Despite this, only a small number of quantitative reviews have been conducted, and the quantitative reviews on ER only included 9 longitudinal studies and they focused on health and work factors (van den Berg et al., 2010), whereas 29 longitudinal studies focused on the relationship between health measures (self-perceived poor health, mental health problems, and chronic disease) and exit from paid employment (van Rijn et al., 2014). Another study explored the association between overweight, obesity, and lack of physical activity and exit from paid employment through disability pension, unemployment, and ER (Robroek et al., 2013). This relative scarcity of meta-analyses on retirement or ER, compared to other research fields, is probably due to the difficulty for retirement researchers to recover enough statistical information from published studies to conduct meta-analytic reviews. Due to the multidisciplinary nature of retirement research and because not every discipline requires researchers to provide these basic statistics in their papers, there are barriers to summarize and systematically review previous findings (Wang, 2013). Therefore, due to its importance in people's lives and for political decisions, conclusions based on meta-analytical findings would be beneficial to determine the links between the antecedent and subsequent correlates of ER.

In this study, meta-analytic techniques are used to summarize an increasing amount of empirical studies in order to provide a solid comprehension of the antecedent and subsequent correlates of ER and to suggest potential avenues for future research. Thus, this meta-analysis has been guided by two research questions. First, what are the main antecedent and subsequent correlates of ER? Second, what methodological and contextual factors would act as potential moderators of the relationship between ER and its antecedent and subsequent correlates? In order to answer these questions, (a) we carried out a meta-analysis of empirical studies on antecedent and subsequent correlates of ER; and (b) we explored potential moderators through analogous ANOVA and weighted regression analysis (random effects model).

## Antecedent correlates of ER

Whereas systematic reviews on retirement- related topics have increased during the last years, only two have been applied to the specific topic of ER. In the first, Feldman (2013) used a general model, the person-environment fit (Edwards et al., 2006), to summarize previous evidence. The person-environment fit theory states that, in order to increase their well-being, individuals try to match their characteristics to those of the environment, considered as their organizations' and groups' requirements and practices. Hence, individuals' congruence with their work environments leads them to remain longer in the job and to rate this environment as more satisfying. When person-environment fit deteriorates, individuals begin seriously considering alternatives, such as ER. As Feldman (2013) stated, fit has been conceptualized as a function of environmental levels such as occupation, organization, job, and group (Kristof-Brown et al., 2002; Vogel and Feldman, 2009).

**Figure 1:**
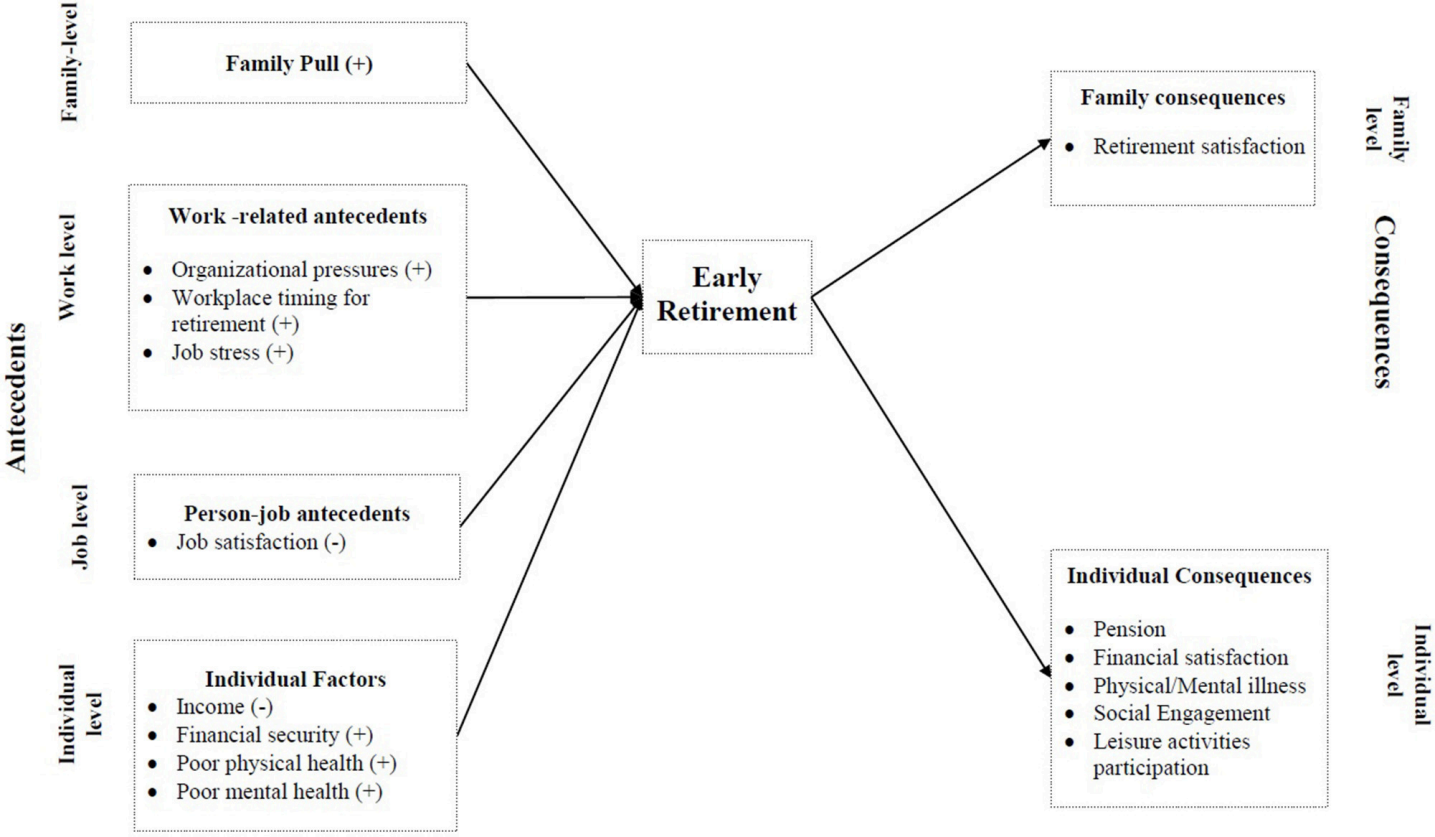
Proposed model for the meta-analysis.

In the second and more recent review, Fisher et al. (2016) integrated previous studies and proposed a specific model to understand the timing of retirement, including antecedent and subsequent correlates grouped in terms of family, work, and individual factors. Based on the life course perspective of retirement (Elder and Johnson, 2001), the model includes a multidisciplinary approach and emphasizes the role of interdependent life spheres (family, work, and community), as well as human agency. This personal power to define and pursue one's goals interacts with the individual constraints under which retirement occurs, such as finances and health. The present review of the antecedent correlates of ER will be conducted from Fisher, Chaffee, and Sonnega' s model, depicted in Figure 1.

## Family-level antecedent correlates

Due to the fact that retirement typically shows an overlap of work and non-work spheres (Wang, 2007); we consider family influences that would attract older workers to ER. First, spouses who have a close relationship would be prone to retire early because they want to spend more time together. Furthermore, a spouse's illness could promote ER for caregiving. This various familial influences should be labeled as *family pull*. From a model based on the theory of planned behavior, van Dam et al. (2009) found that perceived spousal pressure for ER was the strongest predictor. The results obtained by Henkens (1999) suggest that ER of one of the spouses is the result of influence processes within the household, and that ER can be considered, to some extent, a household decision. But at this level, discrepancies have been found, such as those of Friis et al. (2007) and of Kim and Feldman (1998), which showed that participants with a retired or unemployed spouse had higher probabilities of ER, whereas Henkens and Tazelaar (1997) reported only insignificant relationships between having a retired partner and ER (*r* = 0.06). Other results (Kubicek et al., 2010) show that when people (men or women, although it is probably more frequent in men) experience a high level of stress in family life, they may perceive work as a “refuge” to escape from family pressures, so they will avoid ER and postpone the decision to retire as much as possible. Thus, we expect that family pull will be positively related to ER.

## Work-related and person-job antecedent correlates

The firms' policies exert both a direct influence on ER decision, through ER incentives to encourage leaving the job, and an indirect influence through organizational downsizing or pressures toward displacement. On the one hand, there are economic incentives for ER offered by the firms, which are favorable for older workers. On the other, factors such as unsatisfied needs for continued professional training for older employees, managers' negative conceptions of aged workers, and unattractive jobs in terms of task or environmental demands should be considered organizational pressures that precipitate ER. However, empirical studies have provided mixed evidence about these relationships. For instance, Henkens and Tazelaar (1997) showed that organizational restructuring had a low relationship with ER (*r* = 0.08), whereas Fernández Muñoz et al., 2010) found a higher correlation among organizational pressures and ER acceptance (*r* = 0.34), and Alcover et al. (2012) found that organizational pressures was the most important factor for leaving work early. We expect that organizational pressures will be positively related to ER.

Besides being a personal decision, ER is affected by workplace culture, such as older workers' considering the normative time to retire from their occupations. *Workplace timing for retirement* refers to the assessment of how uncommon it is for someone their age to continue working (Feldman, 2013). These evaluations, called “social timing for retirement” (van Solinge, 2006), “subjective norm” (van Solinge and Henkens, 2007), or “social preferences for retirement” (van Dam et al., 2010), predispose people to retire early by facilitating the view of retirement as a normative event. Despite this theoretical consensus, research findings show discrepancies, ranging from *r* = 0.12 between retirement acceptance in the workplace and ER (Whipple, 2001) and *r* = 0.41 between the social norm in the workplace and ER (van Dam et al., 2010). Thus, we expect that workplace timing for retirement will be positively related to ER.

Moreover, job stress would act as a push factor, due to the fact that abandonment of a negative work environment will be experienced as a relief, as supported by empirical evidence (Wang, 2007). Primary studies showed a wide range of results, from Bacharach et al. (2008), who found a relationship between job satisfaction and ER of *r* = 0.20, to Savwoir (1986), who reported negative relationships of *r* = −0.20. Regarding job stress and ER relationships, a great deal of variation has been found in empirical data. For instance, Lund and Villadsen (2005) found that workers exposed to low skill discretion and high conflict were more likely to prefer ER, whereas Fernández Muñoz et al. (2010) observed an insignificant value (*r* = 0.07) for the relationship between job stress and ER.

Also high physical strain was associated with very high ER intention (Sejbaek et al., 2012). The results of the study of von Bonsdorff et al. (2010a,b) showed that poor work ability, frequent emotional exhaustion, low organizational commitment, and low job control were associated with the prevalence of ER intentions among aging employees. Siegrist et al. (2006) found that poor quality of work is significantly associated with intended ER; after adjustment for well-being, odds ratios (OR) of effort-reward imbalance [OR 1.72 (1.43–2.08)] and low control at work [OR 1.51 (1.27–1.80)] on intended ER were observed. However, most of the studies of job-related predictors of ER intention are fragmentary, as they only consider isolated factors such as autonomy, organizational climate, workload, supervisory fairness, or opportunities for career development. One study (Schreurs et al., 2011) attempted to overcome these limitations by using as a theoretical framework, the job demands-resources model, also distinguishing between blue-collar and white-collar workers. Its results showed that the relationship between job resources and work enjoyment is stronger among blue-collar workers, whereas the relationship between job demands and ER intentions is stronger among white-collar workers. Schreurs et al. considered that the use of the job demands-resources model builds on two types of job-related factors (job resources and job demands), and two processes (an energetic and a motivational process) and offered valuable new insights into how the work environment affects the ER decision process. The results obtained by Elovainio et al. (2005) showed that (high) job demands and (low) job control are independent predictors of ER thoughts and offer additional support for the interaction effect of both factors on ER thoughts. Therefore, we expect that job stress will be positively related to ER.

As ER is a crucial event in the employment trajectory, person-job factors are very relevant as antecedent correlates. In this sense, positive attitudes toward the job, such as high satisfaction, would act as pull factors, influencing the individual decision to delay retirement (i.e., Zappalà et al., 2008). Inversely, normal affective organizational commitment was associated with very high ER intention (Sejbaek et al., 2012). Other studies note that work dissatisfaction influences the decision to exit the organization but not the labor market, so there is no direct effect on ER intentions (Perera et al., 2014). We expect that job satisfaction will be negatively related to ER.

Summing up, although a large part of the studies tend to adopt a dichotomous approach to the antecedent correlates of ER (such as health vs. wealth, push vs. pull factors, voluntary vs. involuntary, or own choice vs. no choice), in fact, it seems that the decision is often fraught with shades of ambiguity and involves considerations at several levels of family-, work-, and job-related factors, in addition to individual factors (Robertson, 2000).

## Individual factors

Seminal reviews (Talaga and Beehr, 1989) and a large amount of empirical evidence (e.g., Bazzoli, 1985; Bingefors and Isacson, 2004; Karpansalo et al., 2005; Eberhardt, 2006; Topa et al., 2009; van Dam et al., 2009) supported that, at the individual level, economic and health factors are the most important variables affecting ER.

First, considering that retirement implies a decision related to spending material wealth and savings, it is not surprising that employees with higher incomes will make fewer decisions to retire early (Zappalà et al., 2008). Despite these objective measures of income, subjective measures must also be taken into account (Topa et al., 2011). Financial security would lead to an increased disposition to early withdrawal from the labor market. According to Taylor and Geldhauser (2007), people tend to deny the information about their future financial difficulties in retirement. This bias would account for the different relationships between objective and subjective measures of income, on the one hand, and ER on the other. But even in this pattern of relations between individual factors and ER, there are notable discrepancies in the empirical evidence. Whereas Zappalà et al. (2008) showed that respondents with a better financial situation preferred to postpone retirement, van Dam et al. (2009) reported a positive relationship between adequate financial situation and ER (*r* = 0.21).

Second, most of the primary studies on ER included health, both physical and mental, as a predictor, agreeing that poor health should be considered a powerful determinant of ER (Lawless, 2015). The previous meta-analysis (van den Berg et al., 2010) supported this conclusion, based on general population longitudinal studies. Despite this consensus, primary studies showed inconsistent data. For instance, Leinonen et al. (2016) found (in Finnish older workers) that offering choice about the timing of retirement makes poor health a weaker predictor of ER, because it encourages healthy workers to choose ER regardless of the economic incentives provided to continue working, and Zappalà et al. (2008) only ascertained an irrelevant correlation between health and ER preferences (*r* = −0.06). Thus, regarding economic factors, we expect that income will be negatively related to ER, and financial security will be positively related to ER. Regarding health factors, we expect that both poor physical health and poor mental health will be positively related to ER.

## Subsequent correlates of ER

Whereas various authors have proposed extensive models of the antecedent correlates of ER, the study of its subsequent correlates is still fragmentary, despite its being a topic that very quickly aroused the interest of researchers (Owen and Belzung, 1967). The theory that provides background to review subsequent correlates in a more integrated manner is the referred model of Fisher et al. (2016), which discusses ER outcomes at multiple levels, including individual and familial consequences.

## Individual subsequent correlates

At lower levels, the subsequent correlates of ER can be conceptualized according to individual factors, which primarily include economic, health, and attitudinal facets. Regarding the former, there is prior consensus about the negative relation between ER and objective income, usually assessed through the pension (Blöndhal and Scarpetta, 1998; Blundell et al., 2002). Despite the fact that, for many years, ER plans were designed with very advantageous conditions, the situation has changed progressively (van Solinge and Henkens, 2007). Moreover, at the beginning of retirement, monthly income very probably exceeds the expenses, but after some years, the expenses begin to exceed the income. The negative relation between ER and financial satisfaction has received theoretical support and increasing empirical evidence. Among other reasons, when people rate their available retirement income positively, they suffer from optimistic bias in their financial planning, so they are unhappy when they reach retirement. The literature revealed a noticeable heterogeneity of findings related to satisfaction. Isaksson (1997) showed an increase in global satisfaction during the first two years after ER, whereas other studies failed to find differences (Herzog et al., 1991).

Regarding the relation between ER and health, there is both theoretical and empirical disagreement. Some authors predict that quitting work will lead to an improvement of physical and mental well-being—and even that ER significantly reduces mortality risks (Brockmann et al., 2009)—, whereas others predict harmful effects on health (Ekerdt et al., 1984; van Solinge, 2007; Gallo, 2013)—and even on mortality among male early retirees (Kuhn et al., 2010). Both positions provide supportive empirical results, probably because of the presence of potential moderators, which reveal different patterns or paths to quit the labor market (Wang, 2007). In this sense, other authors note that there is no negative effect of ER on men's health and, if anything, there is a temporary increase in self-reported health and improvements in health in the group of highly qualified workers (Coe and Lindeboom, 2008). Other results (Börsch-Supan and Schuth, 2014) suggest that ER has negative side-effects on the size and intensity of the retirees' social networks, which, in turn, appear to explain part of the accelerated cognitive aging that occurs after ER.

At a broader level, adjustment and well-being of early retirees include social integration and involvement in leisure activities, both considered as retirement adjustment indicators (van Solinge, 2013). Regarding this point, there is an ongoing debate between those who defend that ER will involve the loss of social relations (e.g., Börsch-Supan and Schuth, 2014), primarily made up of co-workers, clients, and colleagues, and those who consider that people will have more time and, therefore, they will cultivate their relationships with family, relatives and friends, and they will engage in more free-time activities (e.g., van Solinge and Henkens, 2007). Moreover, very large discrepancies among the empirical results have been found, probably due to the influence of the moderator factors, which will be analyzed herein. For instance, Robbins et al. (1994) found that social support in ER did not directly influence adjustment but was associated with increases in goal continuity, which, in turn, was linked to increases in leisure quality and life satisfaction. Vaillant (2002) found that retirement was a source of rewards for men who were able to substitute work relationships with other social contacts. Paul and Batinic (2010) found that, although retirees had less social contact (latent function); it was not associated with less psychosocial well-being or worse mental health. However, Rosenkoetter et al. (2001) found that women were less likely to increase participation in social activities than men. Despite the fact that gender as a moderator will be considered in more detail, conclusions based on meta-analytical findings would be beneficial.

## Family-level subsequent correlates

Lastly, regarding attitudinal subsequent correlates, almost all the works that attempt to appraise retirees' adaptation use measures of general satisfaction or satisfaction with some facet of life (van Solinge, 2013). With regard to satisfaction with life in retirement, it is reasonable to consider that a mismatch between expectations and reality is responsible for the negative relation between ER and life satisfaction. Concerning satisfaction with diverse facets of life in retirement, including intentions of re-employing, finances and health again emerge as important aspects (Pattani et al., 2004). There is a debate between those who defend that ER is associated with higher satisfaction with health, either because of a real health improvement or a change in attitude, and those who predict less satisfaction, among other reasons, because health complaints become more salient when people no longer have to think about their work (Gallo, 2013). Existing literature showed a complex pattern of relationships, and there are frequent inconsistencies between the empirical results, allowing us to expect potential benefits from applying meta-analytical techniques.

For individual and family-level subsequent correlates, we examine the relationships between ER, on the one hand, and pension, financial satisfaction, physical/mental illness, social engagement, leisure activities participation, and retirement satisfaction, on the other hand, in an exploratory manner.

## Potential moderator variables

Most of the studies agree in indicating the important influence of moderating variables in the relationships between ER timing and its antecedent and subsequent correlates (Fisher et al., 2016). Firstly, the existence of very discrepant economic and social situations among different countries leads to large differences in those who opt for ER, depending on the origin of the sample included in the study. Variability is high, and some authors have used the classification of the welfare states provided by Esping-Andersen (1999), which distinguishes among Social-democratic and Liberal welfare states. Due to the fact that Social-democratic welfare states grant access to benefits and services based on citizenship, the influence of economic and health factors on ER is expected to be higher. On the contrary, the liberal model is based on private provision, so these citizens need to have strong financial security to take ER.

Secondly, the differences between blue-collar and white-collar workers are not only based on belonging to different social categories, but also on the tasks and features of the job. Accordingly, white-collar workers have better-paid jobs with different physical and psychological demands, so it is therefore reasonable to expect that the relations between economic variables, health, and ER will differ from those of the blue-collar workers (Artazcoz et al., 2010). As a recent study found (Lawless et al., 2015), non-professional occupational status was significantly associated to ER due to disability among Irish people, showing that there are differences in propensity to retire early by socioeconomic groups. Previous research on blue-collar workers (Szubert and Sobala, 2005) showed that perceived negative work conditions (e.g., repetitive tasks, busy work schedules, or heavy lifting at work) were related to increased trends to accept ER programs.

Thirdly, all the studies coincide in pointing out the importance of the voluntary nature of ER and its influence on short- and mid-term outcomes (Shultz et al., 1998). Lack of voluntary decision is associated with loss of control, increase of uncertainty, perception of injustice, and loss of resources, among other underlying processes (Feldman, 2003, 2013; van Solinge, 2006, 2013; Alcover et al., 2012), as well as with a poorer mental health status compared to voluntary retirees (Negrini et al., 2013).

Fourthly, gender and age are two very important sociodemographic characteristics for ER. The former, gender, is associated with a large quantity of economic, health, and cultural factors. First, women have shorter and discontinuous work careers, with lower wages, so they usually have fewer savings for retirement (Taylor and Geldhauser, 2007). Second, higher percentages of men have jobs with physical overload, whereas women tend to experience depression and anxiety more frequently than men (Piccinelli and Wilkinson, 2000). On another hand, women usually develop more than one social role (workers, wives, mothers, caregivers), which will presumably facilitate their engagement in different types of activities when they quit work, thereby influencing the consequences of ER (Dorfman, 2013). In addition, women who show lower levels of work centrality appear to be more inclined to apply for ER, and are generally more satisfied with retirement than males (Isaksson and Johansson, 2000).

With regard to the second characteristic, age can exert influence through its association with other variables. Regarding economic facets of retirement, age will be associated with time in accumulating earnings, and with organizational penalties imposed on those who retire early. Regarding health issues, age will be associated with physical and mental decline.

Lastly, the year of publication may offer relevant information as a proxy for other variables not collected in the investigation, such as the global economic situation or the evolution of the social policies concerning employment and retirement.

Therefore, we examine the moderator role of study location, voluntariness of ER, demographic sample characteristics (occupation, mean age, percentage of males), and study characteristics (year of publication), in an exploratory manner.

## Method

### Literature search and inclusion criteria

Articles published between 1985 and 2016 were identified through computerized databases (*Psycinfo, Medline, ERIC, Academic Search Premier* and *Dissertation Abstracts*) using *early retirement, disability retirement*, and *early retirees* as keywords. Besides, manual searches of journals that publish retirement research have been conducted, using *early retirement* and *early retirees* as keywords. We also contacted informal networks of researchers in the aging-work area by email to request their unpublished papers. This procedure allowed us to obtain two additional manuscripts submitted for publication. We collected a total amount of 471 empirical studies in full text.

**Figure 2:**
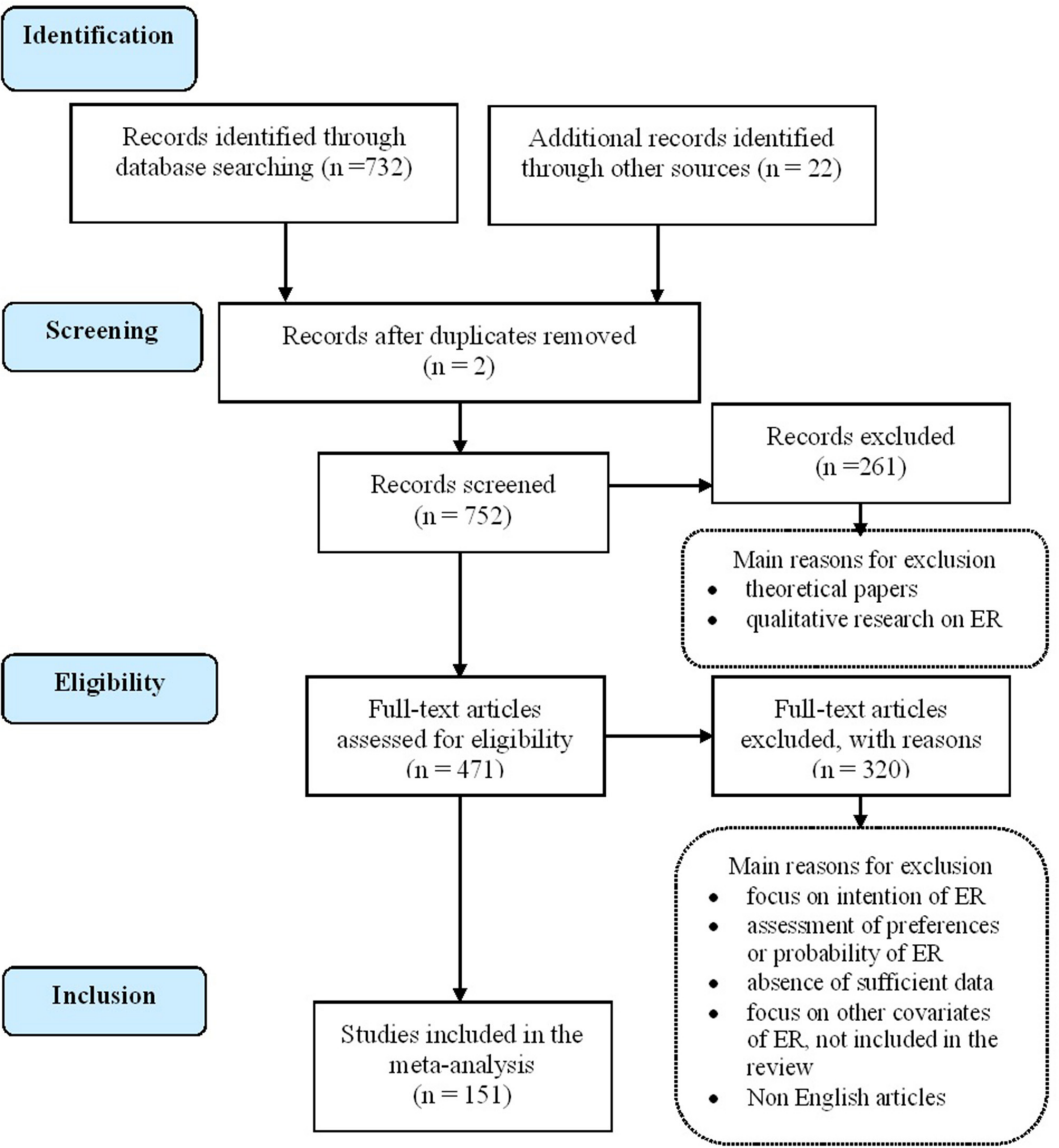
Flow diagram.

The following inclusion criteria have been fulfilled by those papers included in this review: (a) to be an empirical research that includes a sample of early retirees or any empirical measure of ER; (b) to include relationships between ER and its antecedent correlates or its subsequent correlates; (c) to include a Pearson's correlation coefficient or data that would allow us to calculate it. We obtained 151 empirical studies, written in English, French, or Spanish, containing a total amount of 706,937 participants, with a mean sample size of 4,183 participants (*SD* = 12,940.1) and 380 independent effect sizes (ESs), which included 171 independent samples. Both cross sectional and longitudinal papers have been included, as well as those using data from large panel studies. The number of studies screened, assessed for eligibility, and included in the review is provided in the flow diagram (see Figure 2) according to Preferred Reporting Items for Systematic Reviews and Meta-Analyses (PRISMA) recommendations (Moher et al., 2010).

In the phase of record screening, we discarded theoretical papers, qualitative research and those papers devoted to policy recommendations. Then, in a second phase, we screened full text retrieved studies. We discarded those focused on intended ER, subjective expectations, probabilities or preferences for early exit (e.g., Kim and Feldman, 1998; Isaksson and Johansson, 2000; Pienta and Hayward, 2002; Siegrist et al., 2006; Zappalà et al., 2008, among others; Taylor et al., 2014), and studies which their samples partially overlap with another included study. Some studies were excluded due to absence of sufficient data to obtain a Pearson correlation coefficient (Quick and Moen, 1998; Raymo et al., 2011). Additional reasons to discard full text retrieved studies have been the absence of information on the specific relationships between antecedent or subsequent correlates and ER (e.g., Lawless et al., 2015). Non-English articles were included but only if English-language translations were available.

### Coding of studies

Among the ER antecedent correlates, we coded family-level (family pull), work-related (organizational pressures, workplace timing for retirement, job stress, and job satisfaction), and individual factors, both economic and health, (income, financial security, poor physical health, poor mental health). Among the ER subsequent correlates, we coded family-level factors (retirement satisfaction), and individual subsequent correlates [economic, health and attitudinal factors (pension, financial satisfaction, mental/physical illness, involvement in leisure activities, and social participation)]. As previous studies have shown different patterns of relationships between objective and subjective measures of income and antecedents and consequences (Taylor and Geldhauser, 2007), we coded these factors separately. We considered *income* as objective income, whereas *financial security* included affectively loaded measures such as financial concerns, among others.

Six potential categorical and continuous moderating variables were coded: (a) *methodological variables* of the sample: participants' occupational categories (white collar, blue collar), participants' perception of ER (voluntary, forced and disabled), gender of the sample (more or <50% of males) and participants' age (over 55 years, under 55 years); and (b) *contextual variables*: study location (North America, European Union, other) and year of publication or completion. The study location was then used to conduct analyses as a function of the welfare states' classification. Due to the fact that the structure of the State and its economic basis have strong relationships with the conditions under which ER is taken, the study location brings the opportunity to consider this data as moderator variable. The process was carried out by two independent raters who, after being trained in the procedure, analyzed 40% of the studies and reached an acceptable agreement level (*r* = 0.85). The coding manual has been reviewed when discrepancies arise in order to solve them. In order to ensure independence of ES, we retained only one ES per sample. When a single study included two or more independent samples (e.g., public vs. private employees, men vs. women, voluntary vs. involuntary retirees), we included their results as independent studies (e.g., Shultz et al., 1998).

In this meta-analysis, the ES was Pearson's correlation coefficient (*r*). ES values have been treated with Comprehensive Meta-analysis 2.0 (CMA; Borenstein et al., 2005) in order to be converted to Fisher's *Z* transformation of *r*. The guideline to interpret the magnitude of ES was *r* < 0.20 = low ES value, *r* between 0.20 and 0.30 = medium ES value, and *r* > 0.30 = high (Hemphill, 2003: 78). The 95% confidence interval was we also reported. Homogeneity analyses were carried out with *Q* statistics (Hunter and Schmidt, 1990). Due to its shortcoming of poor power when a small number of studies are included, we provide *I*^2^. The *I*^2^ index can be interpreted as the percentage of the total variability in a set of ESs due to true heterogeneity (Borenstein et al., 2015). We analyzed the influence of moderator variables using a categorical model (analogous-ANOVA) and weighted regression analysis (mixed effects model), as recommended by Quintana (2015). Regarding publication bias, different indices are provided. Publication bias occurs because unpublished research with non-significant findings is less available and because journals' practices tend to favor studies with significant findings. As inspection of funnel plot asymmetry (Sterne et al., 2005) seems to be insufficient, different indices should be used to examine the presence of publication bias. In the present study, we included the more classic Fail Safe N proposed by Rosenthal (1979), the Rank correlation test proposed by Begg and Mazumdar (1994), Egger's (Egger et al., 1997) Test of the intercept and Duval and Tweedie's (2000) Trim and Fill test.

We did not exclude studies as outliers for two reasons. First, there is no agreement on the percentage of ES that should be excluded (Hedges and Olkin, 1985). Second, the exclusion of outliers made it problematic to test for differences among categories of moderator variables.

## Results

### Description of studies

The 151 studies have been performed or published before 2016 (11 between 1985 and 1995, 11 between 1996 and 2000, 31 between 2001 and 2005, and 98 between 2006 and 2014). Most studies were conducted in the European Union (104) and in the United States of America (39), and the remaining studies included participants from Asia, Australia, or Canada. The mean age of the sample was 56.68 years (*SD* = 7.77). The mean percentage of males in the samples was 60.81% (*SD* = 35.67).

### Antecedent correlates of ER: mean ES

Family pull exerted a positive influence on ER, reaching an average low level ES (*r* = 0.19, CI [0.09, 0.29]). Both organizational pressures and workplace timing for retirement exhibited positive relationships with ER (Table 1), with workplace timing for retirement showing an average ES of medium level (*r* = 0.25, CI [0.17, 0.33]), and organizational pressures (*r* = 0.22, CI [0.15, 0.29]) reached a medium level ES. The correlation was much larger for the workplace timing-ER relationship than for the organizational pressures-ER, despite the fact that their CIs substantially overlapped. Job stress reached a positive mean low level ES (*r* = 0.16, CI [0.12, 0.20]), with a lower level size. Job satisfaction-ER relationships were negative, with lower levels values (*r* = −0.16, CI [−0.20, −0.11]).

**Table 1:** Mean weighted effect sizes for meta-analysis.

**Variables**	***k***	**Weighted *r***	***SD***	**95% CI**		**Q (df)**	***I*^2^**
				**Ll**	**Ul**		
**ANTECEDENT CORRELATES**							
**Family-level antecedent correlates**							
Family pull	19	0.19	0.05	0.09	0.29	739.13 (18)^***^	97.5
**Work-related antecedent correlates**							
Organizational pressures	20	0.22	0.04	0.15	0.29	864.22 (19)^***^	97.8
Workplace timing for retirement	9	0.25	0.04	0.17	0.33	99.2 (8)^***^	91.9
Job stress	27	0.16	0.02	0.12	0.20	391.5 (26)^***^	93.4
**Person-job antecedent correlates**							
Job satisfaction	27	−0.16	0.02	−0.20	−0.11	851.68 (26)^***^	96.9
**Individual factors**							
Income	24	−0.13	0.03	−0.18	−0.07	2, 298.7 (23)^***^	98.9
Financial security	27	0.22	0.03	0.16	0.28	585.75 (26)^***^	95.56
Poor physical health	109	0.20	0.02	0.16	0.24	23, 669.9 (108)^***^	99.5
Poor mental health	41	0.20	0.02	0.15	0.25	864.6 (40)^***^	95.37
**SUBSEQUENT CORRELATES**							
**Individual subsequent correlates**							
Pension	20	0.18	0.05	0.07	0.29	1, 861.68 (19)^***^	98.98
Financial satisfaction	11	−0.15	0.05	−0.24	0.06	73.63 (10)^***^	86.42
Mental/Physical Illness	12	0.08	0.05	−0.02	0.18	307.37 (11)^***^	96.42
Social engagement	4	−0.25	0.11	−0.47	−0.04	27.67 (3)^***^	89.16
Leisure activities	8	0.12	0.05	0.03	0.20	66.26 (7)^***^	89.44
**Family-level subsequent correlates**							
Retirement satisfaction	10	0.12	0.14	−0.16	0.39	1, 837.51 (9)^***^	98.77

k, number of correlations; 95% CI, 95% confidence interval around Weighted r; Ll, lower limit; Ul, upper limit. Q (df), chi square test for homogeneity (degrees of freedom); I^2^, percentage of variance beyond the sampling error.

*** *p < 0.001*.

Finally, income-ER exhibited a negative relationship, with a mean lower level ES of *r* = −0.13. At the same time, financial security reached a medium level ES (*r* = 0.22, CI [0.16, 0.28]). A positive relationship between previous poor health, both physical (*r* = 0.20, CI [0.16, 0.24]), and mental (*r* = 0.20, CI [0.15, 0.25]) was obtained (see Table 1).

### Subsequent correlates of ER and mean ES

We expected significant relationships between ER and both economic and health subsequent correlates. A mean ES of low level for the relationship between ER and pension (*r* = 0.18, CI [0.07, 0.29]) was obtained, whereas the relationship between ER and financial satisfaction showed also a lower level ES (*r* = −0.15, CI [−0.24, 0.06]), and their CIs did not substantially overlap. The relationship between illness and ER only reached a lower level ES (*r* = 0.08, CI [−0.02, 0.18]). Our findings showed a medium mean ES for social engagement (*r* = −0.25, CI [−0.47, −0.04]), and a lower ES for participation in leisure activities (*r* = 0.12, CI [0.03, 0.22]). And, finally, a lower level ES (*r* = 0.12, CI [−0.16, 0.39]) was also reached for retirement satisfaction.

### Publication bias analyses

Firstly, related to publication bias, Classic Fail Safe N, which is large in all of our meta-analyses, allows us to feel confident that the treatment effect, while possibly inflated by the exclusion of some studies, is nevertheless not null. Despite the fact that researchers stated that Fail-Safe N exceeding five times the number of included effects would indicate robust effect, we acknowledge that some of our meta-analyses have a small *k*, and we continued exploring potential bias by additional tests. Secondly, we applied Begg and Mazumdar's (1994) Rank correlation test, which provides Kendall's tau b-value, and which suggests that publication bias exists if the associated *p*-value is statistically significant (*p* < 0.05). With this test, we found that the job stress-ER and poor physical health-ER meta-analyses might be biased (see Table 2).

**Table 2:** Publication biasindices.

**Variables**	**Classic fail-safe *N***	**Rank correlation test (Kendall's tau b; *p*-value^a^)**	**Egger's test**	**Duval and tweedie's trim and fill (Random effects model point estimate and 95% CI)**
**ANTECEDENT CORRELATES**				
**Family-level antecedent correlates**				
Family pull	1,398	0.70; *p* = 0.34	Intercept = 3.67; *t* = 1.58; *df* = 17; *p* = 0.06	0.04 (−0.07; 0.16)
**Work-related antecedent correlates**				
Organizational pressures	2,734	0.06; *p* = 0.36	Intercept = 5.57; *t* = 2.96; *df* = 18; *p* = 0.001	0.05 (−0.03; 0.13)
Workplace timing for retirement	984	0.36; *p* = 0.09	Intercept = 4.76; *t* = 1.39; *df* = 7; *p* = 0.10	0.19 (0.10; 0.28)
Job stress	3,824	0.26; *p* = 0.03	Intercept = 3.14; *t* = 2.71; *df* = 25; *p* = 0.00	0.07 (0.03; 0.12)
**Person-job antecedent correlates**				
Job satisfaction	7,105	−0.09; *p* = 0.25	Intercept = −1.62; *t* = 0.87; *df* = 25; *p* = 0.20	Values are unchanged.
**Individual factors**				
Income	2,348	0.05; *p* = 0.35	Intercept = −4.03; *t* = 1.87; *df* = 22; *p* = 0.04	Values are unchanged.
Financial security	4,461	0.12; *p* = 0.19	Intercept = 3.03; *t* = 2.31; *df* = 25; *p* = 0.02	0.10 (0.03; 0.17)
Poor physical health	988,789	0.36; *p* = 0.00	Intercept = −6.31; *t* = 4.76; *df* = 106; *p* = 0.00	Values are unchanged.
Poor mental health	8,288	−0.12; *p* = 0.14	Intercept = 2.33; *t* = 2.38; *df* = 39; *p* = 0.01	0.12 (0.07; 0.17)
**SUBSEQUENT CORRELATES**				
**Individual subsequent correlates**				
Pension	4,063	−0.06; *p* = 0.72	Intercept = 0.21; *t* = 0.05; *df* = 18; *p* = 0.48	Values are unchanged.
Financial satisfaction	222	0.13; *p* = 0.29	Intercept = 1.24; *t* = 0.58; *df* = 9; *p* = 0.29	Values are unchanged.
Mental/physical illness	428	−0.05; *p* = 0.42	Intercept = −1.58; *t* = 0.79; *df* = 10; *p* = 0.22	Values are unchanged.
Social engagement	55	0.50; *p* = 0.15	Intercept = 8.28; *t* = 0.98; *df* = 2; *p* = 0.22	−0.33 (−0.54; −0.12)
Leisure activities	43	−0.04; *p* = 0.45	Intercept = 3.26; *t* = 3.35; *df* = 6; *p* = 0.007	−0.02 (−0.09; 0.06)
**Family-level subsequent correlates**				
Retirement satisfaction	305	0.40; *p* = 0.05	Intercept = 4.25; *t* = 0.70; *df* = 8; *p* = 0.25	−0.06 (−0.33; 0.21)

a *p-value based on continuity-corrected normal approximation*.

Thirdly, as this approach is still limited, due to the fact that its power is moderate for smaller meta-analyses, we used an alternative test. Egger's (Egger et al., 1997) test is best suited for smaller meta-analyses (*k* < 25 studies). This test uses a regression model to predict the standardized ES, and the intercept of the regression is the bias. Consequently, a *t*-value, with a *p* < 0.001 is offered as an indicator of potential publication bias. When applying this procedure, we found that the organizational pressures-ER, job stress-ER, poor physical and poor mental health-ER, and leisure activities -ER meta-analyses would be affected by publication bias. Finally, when there is evidence of publication bias, a method can be used to adjust the meta-analytical estimations and provide a reasonable estimate of how many studies are missing. The Trim and Fill method may slightly overestimate missing studies, but it can be used to assess the potential impact of these probable studies on the ES (Quintana, 2015).

According to these findings, we contend that the point estimate under the random effects model would be smaller for seven meta-analyses (Family Pull, Organizational pressures, Workplace Timing for retirement, Job stress, Financial security, Poor mental Health, Leisure activities participation, and Retirement satisfaction). These point estimates should not be used to support any conclusion, as, based on these previous analyses, we consider that publication bias is a potential concern with these data. Despite this fact, Sterne et al. (2005) acknowledge that these analyses may be inaccurate when there is substantial between-study variability.

### Potential moderator variables

Table 1 shows the results for the *Q* statistics analyses and *I*^2^. Both values revealed significant heterogeneity across studies, with percentages of explained variance due to this heterogeneity ranging from 86% for the ER-financial satisfaction relationship to 99.6% for poor physical health-ER. In view of these results, we explored potential moderator variables using a categorical model (analogous ANOVA) and weighted regression analysis (random effects model).

Table 3 shows the results of moderator analyses as a function of the origin of the sample. We expected that welfare states would have effects on individual factors-ER relationships. Regarding the income-ER relationship, the correlation was much larger for studies from countries with a Social-democratic welfare state than for those from Liberal welfare states, despite the fact that their CIs substantially overlapped. Results showed that financial security ES seems to be similar for participants from Liberal and Social democratic welfare states. Regarding the poor physical health-ER relationship, both *Q*_(*b*)_ and *Q*_(*w*)_ reached statistical significance, showing that a greater level of heterogeneity remained unexplained by the analyses. The mean ES for each category showed higher values for Social-democratic welfare states, despite that the CIs substantially overlapped. Moreover, considering the poor mental health-ER relationship, the mean ES distinguished participants from Liberal welfare states, with higher values than those from Social-democratic welfare states. Finally, regarding ER-pension relationships, the mean ESs showed lower values for participants from countries with Liberal welfare states.

**Table 3:** Weighted analysis of variance as a function of origin of the sample.

**Variables**	***Qb (df)/Qw (df)***	**Effect size (Ll/Ul)**	
		**Liberal**	**Social democratic**
**ANTECEDENT CORRELATES**			
Income-ER	74.6 (1)^***^/2,224.1 (22)^***^	−0.08 (−0.14/−0.03)	−0.17 (−0.27/−0.07)
Financial security-ER	8.55 (1)^***^/577.21 (25)^***^	0.22 (0.13/0.31)	0.22 (0.13/0.32)
Poor physical health-ER	12,010.7 (1)/11,511.7 (105)^***^	0.16 (0.07/0.25)	0.23 (0.19/0.25)
Poor mental health-ER	48.52 (1)^***^/816.1 (39)^***^	0.18 (0.09/0.28)	0.21 (0.16/0.26)
**SUBSEQUENT CORRELATES**			
ER-Pension	297.2 (1)^***^/1,564.4 (17)^***^	0.10 (−0.08/0.29)	0.25 (0.15/0.35)

Qb (df)/Qw: chi square test for homogeneity between and within (degrees of freedom);

*** *p < 0.001*.

Related to the participants' labor status, the ES showed differences between blue- and white-collar workers both in the individual-ER and in the job stress-ER relationships. Despite the fact that their CIs substantially overlapped, our results showed that the negative correlation between income and ER was much larger for studies with blue-collar workers than for white-collar workers. Both *Q*_(*b*)_ and *Q*_(*w*)_ reached significant values for the financial security-ER relationship, and similar ES values were reached for blue-collar workers as for white-collar workers. We expected differences in the relationship between poor physical health-ER among blue- and white-collar retirees, and the results provided support for this expectation. Although not addressed in our predictions, we observed that poor physical health effects on ER were moderated by participants' occupational categories (*r* = 0.16 vs. *r* = 0.22 for white- vs. blue-collar). Regarding the ER-pension relationships, our findings showed that the ES was much larger for studies with white-collar participants than for those with blue-collar workers (see Table 4).

**Table 4:** Weighted analysis of variance as a function of participants' occupational categories.

**Variables**	***Qb (df)/Qw (df)***	**Effect Size (Ll/Ul)**	
		**White collar**	**Blue collar**
**ANTECEDENT CORRELATES**			
Job stress-ER	79.7 (1)^***^/311.9 (25)^***^	0.14 (0.09/0.19)	0.19 (0.13/0.25)
Income-ER	38.74 (1)^***^/2,259.9 (22)^***^	−0.08 (−0.19/−0.02)	−0.15 (−0.22/−0.07)
Financial security-ER	16.6 (1)^***^/569.03 (24)^***^	0.22 (0.15/0.29)	0.20 (−0.09/0.51)
Poor physical health-ER	595.3 (1)^***^/23,049.7 (105)^***^	0.16 (0.08/0.24)	0.22 (0.17/0.26)
**SUBSEQUENT CORRELATES**			
ER-Pension	141.61 (1)^***^/1720.07 (18)^***^	0.32 (−0.15/0.80)	0.15 (0.06/0.24)

Qb (df)/Qw: chi square test for homogeneity between and within (degrees of freedom);

*** *p < 0.001*.

We expected that voluntariness of ER would play a moderator role in the individual factors-ER and in the ER- subsequent correlates relationships. Table 5 shows the results of the analogous ANOVA as a function of the perception of forced retirement. Disabled workers were considered a different category, and data are provided when we found sufficient information among the primary studies. Regarding antecedent correlates of ER, the findings showed higher values for the negative income-ER relationship among forced retirees than for voluntary ones, despite the fact that both *Q*_(*b*)_ and *Q*_(*w*)_ reached statistical significance. The effect of financial security on ER was moderated by voluntariness, such that a small ES was shown for forced retirees whereas a large ES was exhibited for voluntary retirees.

**Table 5:** Weighted analysis of variance as a function of participants' perception of forced retirement.

**Variables**	***Qb (df)/Qw (df)***	**Effect size (Ll/Ul)**		
		**Voluntary**	**Forced**	**Disabled**
**ANTECEDENT CORRELATES**				
Income-ER	33.9 (2)^***^/2,264.7 (21)^***^	−0.04 (−0.10/0.02)	−0.16 (−0.23/−0.9)	−0.02 (−0.07/0.04)
Financial security-ER	120.9 (1)^***^/464.8 (24)^***^	0.34 (0.05/0.62)	0.19 (0.14/0.24)	−0.07 (−0.45/0.32).
Poor physical health-ER	914.31 (2)^***^/22,752.7 (105)^***^	0.20 (0.16/0.25)	0.05 (−0.07/0.17)	0.26 (0.14/0.37)
Poor mental health-ER	0.53 (2)/864.10 (34)^***^	0.23 (0.17/0.28)	0.11 (0.002/0.23)	0.07 (−0.07/0.21).
**SUBSEQUENT CORRELATES**				
ER-Pension	284.79 (1)^***^/1,576.9 (18)^***^	0.20 (0.09/0.30)	−0.17 (−0.21/−0.13)	n. a.
ER-Retirement satisfaction	0.58 (1)/272.85 (4)^***^	0.24 (−0.14/0.63)	−0.10 (−0.97/.073)	n. a.

Qb (df)/Qw: chi square test for homogeneity between and within (degrees of freedom);

*** *p < 0.001; n. a., not available*.

In the case of poor physical health, both *Q*_(*b*)_ and *Q*_(*w*)_ reached significance, and the higher value was shown by disabled retirees. As Table 5 shows, the larger ES was exhibited for disabled retirees than for voluntary and forced ones, despite that the values were similar and the CIs substantially overlapped. Related to poor mental health, the data showed larger values for voluntary retirees than for forced or even for disabled ones.

We expected that gender would play a moderator role in the individual factors-ER relationships. On the one hand, our findings showed that ES was larger for males than for females in the income-ER relationships but not in the financial security-ER relationships. On the other hand, females showed a stronger ES for poor health, both physical and mental, than males. Regarding subsequent correlates of ER, males exhibited higher values than females in the ER-pension relationships (see Table 6).

**Table 6:** Weighted analysis of variance as a function of participants' gender.

**Variables**	***Qb (df)/Qw (df)***	**Effect Size (Ll/Ul)**	
		**Females**	**Males**
**ANTECEDENT CORRELATES**			
Income-ER	420.9 (1)^***^/1,877.7 (22)^***^	−0.09 (−0.14/−0.03)	−0.17 (−0.38/0.05)
Financial security-ER	49.5 (1)^***^/536.3 (25)^***^	0.24 (0.14/0.35)	0.18 (0.12/0.25)
Poor physical health-ER	1,807.39 (1)^***^/21,859.7 (106)^***^	0.21 (0.16/0.26)	0.19 (0.14/0.24)
Poor mental health -ER	6.09 (1)^*^/858.5 (39)^***^	0.22 (0.16/0.29)	0.17 (0.09/0.24)
**SUBSEQUENT CORRELATES**			
ER-Pension	14.48 (1)^***^/1,847.2 (18)^***^	0.15 (0.02/0.27)	0.20 (−0.001/0.39)

Qb (df)/Qw: chi square test for homogeneity between and within (degrees of freedom);

* p < 0.05.

*** *p < 0.001*.

We expected that participants' age would act as a moderator both of the individual factors-ER and the ER- subsequent correlates relationships. Our findings showed that studies with participants aged over 55 years obtained larger ESs in the income-ER relationships, compared with those with participants aged below 55 years. This pattern of results was also exhibited for the financial security-ER relationship. Regarding poor physical health, participants aged over 55 years showed smaller ESs than younger participants. For the poor mental health-ER relationship, the opposite was observed. Finally, regarding ER-pension relationships, our findings exhibited larger ES values for younger participants than for older respondents (see Table 7).

**Table 7:** Weighted analysis of variance as a function of participants' age.

**Variables**	***Qb (df)/Qw (df)***	**Effect size (Ll/Ul)**	
		**Below 55 years**	**Over 55 years**
**ANTECEDENT CORRELATES**			
Income-ER	372.4 (1)^***^/1,926.3 (22)^***^	−0.05 (−0.11/0.02)	−0.19 (−0.32/−0.05)
Financial security-ER	1.41 (1)/584.2 (24)^***^	0.17 (0.12/0.22)	0.24 (0.13/0.36)
Poor physical health-ER	12,888.1 (1)^***^/10,778.9 (106)^***^	0.22 (0.18/0.25)	0.18 (0.11/0.25)
Poor mental health -ER	226.01 (1)/638.6 (39)^***^	0.19 (0.16/0.26)	0.21 (0.16/0.27)
**SUBSEQUENT CORRELATES**			
ER-Pension	20.61 (1)/1,841.07 (18)^***^	0.24 (0.03/0.46)	0.15 (0.01/0.28)

Qb (df)/Qw: chi square test for homogeneity between and within (degrees of freedom);

*** *p < 0.001*.

Weighted regression analyses were used to test the influence of the unique quantitative variable, year of publication. We expected that relationships between antecedent correlates-ER and ER- subsequent correlates would be affected by year of publication. The results provided mixed support for this prediction. Considering year of publication, the results revealed a highly significant *Q*_*E*_ in many cases (income-ER, poor physical health-ER, poor mental health-ER, and ER-pension) and, in contrast, *Q*_*R*_ failed to reach significant values. As a consequence, the *R*^2^ analog values were negligible in these analyses. Regarding financial security-ER, results seem to be more promising (*R*^2^ = 0.10), despite that only *Q*_*E*_ was statistically significant (see Table 8).

**Table 8:** Weighted multiple regressions as a function of year of publication.

**Variable**	**Moderator: year of publication**		
	***R*^2^ analog**	***Q*_R_ (*df*)**	***Q*_E_ (*df*)**
**ANTECEDENT CORRELATES**			
Income- ER	0.01	0.33 (1)	1,603.4 (22)^***^
Financial security- ER	0.10	2.72 (1)	555.89 (25)^***^
Poor physical health- ER	0.00	0.07 (1)	23,622.02 (106)^***^
Poor mental health- ER	0.03	0.81 (1)	862.18 (39)^***^
**SUBSEQUENT CORRELATES**			
ER- Pension	0.00	0.82 (1)	1,614.21 (18)^***^

*** *p < 0.001*.

## Discussion

The present meta-analysis was aimed to synthesize the results of the empirical studies of ER and to meta-analytically test a theoretical model of its antecedent and subsequent correlates derived from the Fisher et al. (2016) Model. The procedure has been developed in three steps: first, separate meta-analyses of ER- its antecedent and subsequent correlates have been conducted. Second, analyses of potential moderating variables have been conducted in order to offer some explanation for the variability between empirical findings. Some analyses were based on a restricted number of primary researches, but the results allowed us to reach several conclusions based on the empirical findings. Our findings can be seen as a research tool that allows us to highlight the overall network of relationships between constructs and to improve our understanding of the retirement process.

What are the main antecedent and subsequent correlates of ER?

First, the weight of the social-normative expectations of ER clearly emerge (Feldman and Beehr, 2011), due to the fact that the variables that entail external pressures from any source showed higher ESs. All things considered, workplace timing of retirement is the best predictor of ER, followed by organizational pressures. Although there are a number of individual, family, and macro-economic and sociocultural factors that are antecedent correlates of retirement timing, work- and organizational-related factors are two of most relevant (Fisher et al., 2016). Our results confirm previous research, which showed that older workers' social environment (workplace timing and organizational pressures) is related to ER (van Solinge and Henkens, 2007).

These findings underline the relevance of an organizational climate for successful aging for older people's motivation to continue working (Zacher and Yang, 2016). In this sense, if the organization provides a climate for successful aging, older employees would perceive better job opportunities, and these perceptions, in turn, would be positively related to affective commitment and would discourage them from ER (Armstrong-Stassen and Schlosser, 2008). In the same line, as a recent study stated (Segura et al., 2017), lack of a healthy environment at work, less autonomy, task significance, variety, and feedback could derive in a loss of meaningfulness, responsibility, and knowledge for older workers, which could lead to seeking ER. Despite the fact that there are some workers' strategies, such as optimization, which could serve as an additional source of motivation or even as a buffer, it seems very difficult to reduce the negative impact of the unhealthy environment on employee ER.

Second, a cascade effect of person-environment fit levels has been shown, beginning with organizational fit and following with family fit, as various authors suggested (Kristof-Brown et al., 2002; Feldman, 2013). Considering the degree of impact on ER, workplace timing preferences was the first variable, organizational pressures the second, and family pull the last. Although an individual's perception of push and pull factors occurs in context, and although an individual's perception of context can lead to the same event being rated as either a push or a pull by different workers (Shultz et al., 1998), it seems that a pattern of relationships exists, as Feldman (2013) suggested. This finding supports the evidence about the impact of positive job characteristics on intended retirement time (Ecklund, 2014), as well as the fact that, although people cannot control the external circumstances affecting the retirement process, they can at least anticipate the conditions of the process by retirement planning and preparation programs (Palaci et al., 2017).

Third, when comparing family-level and person-job antecedent correlates, we realized that poor adjustment between the older workers and their immediate environment has the strongest impact on ER. Family pull, which represents an immediate social cue, exerted more influence on ER than did job satisfaction. Regarding this pattern, we must take into account that ER not only implies losing social relations made up of colleagues and supervisors, but it also offers the possibility of gaining stronger contacts with family. Hence, family and partner involvement are very important to deal with retirement transition (Feldman, 2013), as supported by recent evidence (Topa et al., 2017a; Yeung and Zhou, 2017).

Fourth, regarding ER antecedent correlates at the work/job level, job satisfaction showed the same ES as job stress. As older workers' preferences change from extrinsic to intrinsic rewards, ES in the job satisfaction-ER relationship shows that emotional job attachment would have a similar influence on ER decisions as the job's negative characteristics, which, in turn, would trigger a stress process. As some researchers have recently shown, a work environment with many organizational resources will foster work engagement, which in turn will increase the likelihood of job satisfaction and the intention to stay longer (Guglielmi et al., 2016). But this relationship was shown to be moderated by the joint influence of job demand intensity and age, confirming that different patterns emerge as a function of employees' age (Topa et al., 2016), and socioeconomic characteristics (Davies et al., 2017).

Fifth, within individual ER antecedent correlates, health seems to exert a great influence. Our results also suggest that both physical and mental health have the same impact on ER. The experience of preretirement work stress and a poor psychosocial quality of work (Siegrist et al., 2006) may be related to the perception of poorer health, both physical and mental, such that both act as push factors to ER. Additionally, health seems to exert more influence than objective measures of finances—income—but not than perceptual measures of financial security. However, it is necessary to take into account that the relationships between health and finances are very complex. Firstly, illness can affect subjective perceptions of income adequacy. This would be due to the fact that these measures were affectively loaded, and poor physical and mental health could bias them (Topa and Herrador-Alcaide, 2016). Secondly, health could affect income, due to the fact that illness limits access to better jobs and makes saving more difficult. Lastly, it has been suggested that combinations of stressful events such as financial difficulties can make people more vulnerable when a negative event occurs. In this light, income may be less important for healthier people than for those who have suffered a decline in health.

Regarding economic antecedent correlates of ER, objective measures of income showed less influence than subjective ones. As previous researchers suggested, it is likely that people earning high salaries seek to delay their retirement in order to accumulate earnings that allow them financial freedom (Zappalà et al., 2008), as shown by the negative ES of income. Due to their nature, objective indices of finances may serve as better predictors of the ability to meet financial needs after retirement, whereas affective-loaded measures would be biased by unrealistic optimism about retirement, as indicated by the positive and stronger ES of financial security. These findings are in line with recent studies that underline the influence of some psychological features that could bias economic information processing (Topa et al., in review). Our results confirm that these phenomena could be related to each other despite that there are not interchangeable. The complexity of our findings highlights the statement of Feldman (2003, p. 281) that “treating the retirement decision as one made solely by the individual on the basis of the earnings may miss a considerable amount of variance in the decision-making process.”

Our findings related to the subsequent correlates of ER showed two controversial relations with illness, on the one hand, and pension, on the other, which both remained positive in our meta-analytical results. Although the debate between defenders of the positive and negative effects of ER on health exceeds the scope of this review, our findings provide evidence of the underlying processes. As empirical studies stated (Paul and Batinic, 2010), ER would entail a deterioration of resources that ensure stability, status, and privileged conditions; and of valuable personal resources-self-esteem or social skills-which, and in turn, could have an impact on health (Topa et al., 2017b; Yeung and Zhou, 2017). But, despite the fact that this review could not include a statistical control of the negative influence of previous poor health on later illness—which should be done by a meta-analytic structural equation model—this pattern would exist. Recent research based on a longitudinal study showed a long-term impact of pre-retirement depression on post-retirement depression, besides its influence through the perception of threats during the transition (Topa and Valero, 2017). Does ER have a fatal attraction? As Kuhn et al. (2010) found, death causes indicate a significantly higher incidence of cardiovascular disorders among male early retirees, suggesting that changes in health-related behavior explain increased mortality among males, although they did not find any adverse effect of ER on mortality for females. Although ER has gained an increasingly positive image, the data of this review do not support such an optimistic view.

Regarding the economic subsequent correlates, objective measures—pension—showed a higher impact than subjective ones—financial satisfaction—, contrary to the pattern exhibited in the antecedent correlates section, but these findings must be deeply analyzed in order to detect differences as a function of voluntariness and labor status. Again, it is necessary to recognize that illness and finances have mutual influences, and both factors can reduce quality of life in retirement. In this sense, chronic illness can negatively influence satisfaction with income in retirement, not only because it conditions needs assessments, but also because it can eat into the savings actually set aside by the more farsighted retirees, who may see their resources diminished by the rising cost of health services. And, in turn, poor health status and pessimistic financial expectations predict greater household financial distress (Litwin and Sapir, 2009).

Our findings related to the subsequent correlates of ER indicate that the strongest negative impact was on social engagement, which has the highest ES among the ER subsequent correlates. Although maintaining social contact may not only depend on work-related social relations, one of the factors with the greatest influence on the disposition to ER is the expectation of social contact (Henkens and Tazelaar, 1994). Our results indicate that, even considering this expectation, early retirees undergo a significant reduction of social integration, a negative experience that does not seem to be compensated through non-work activities such as volunteering, family duties, and hobbies (Kim and Feldman, 2000).

Turning to leisure activity participation, the ER impact was positive but low. Perhaps this finding would be a consequence of global changes in volunteerism and other forms of leisure activities. Due to the fact that NGOs seem to gradually lose their position and meaning as an exclusive place for social participation through volunteerism, early retirees, and other volunteers only develop weak and unstable relations with their organizations. Hence, volunteers increasingly prefer individual, project-based tasks, they frequently switch organizations and, therefore, they are currently more oriented to a relational type of psychological contract with their NGOs, as recent studies suggest (Aranda et al., 2017).

Finally, the significant influence of ER on retirement satisfaction suggested that some of the concerns about the possible negative effects of ER are exaggerated and partially an artifact of the causes of ER. As Hobfoll's (2011) conservation of resources theory stated, people's well-being is rooted in access to a reserve of valuable resources, which cover basic needs and serve as an expression of worth and status, as well as providing protection against future losses. While work is associated with a broad spectrum of such resources, ER could be associated with others, such as freedom, leisure time, and affects. Furthermore, as recent studies report, not only the influence of available resources near to retirement on satisfaction after the event should be considered but also the influence of distal antecedent correlates on resources (Earl et al., 2015; Topa and Pra, 2017). Despite this fact, retirement can also provoke unease, gloom, and dread, and can be associated with fear of death, a complex emotional phenomenon that includes apprehension both for oneself and for loved ones. Empirical evidence shows that fear of death impinges on most spheres of life, specifically on long-term decisions, but only recent studies begin to consider the role of fear of death in explaining retirement planning (Topa et al., in review).

What methodological and contextual factors would act as potential moderators of the relationship between ER and its antecedent and subsequent correlates?

Extending prior research that started to examine moderators of ER-antecedent links, we meta-analytically compared studies conducted under different welfare regimes, finding that effects of objective financial measures were stronger for social democratic welfare states, whereas subjective finances impact the most on participants from liberal countries. As social democratic welfare states provide access to services based on citizenship, poor physical and mental health both led to ER, showing greater ESs for those countries than for liberal welfare states. As Feldman (2013) warned, these results also seem to confirm the possible interference of third variables in the relations between antecedent correlates and ER. Further analyses would show that differences between professional sectors emerge, revealing still more heterogeneity in the same country. As the Fisher et al. (2016) Model proposed, several social factors indirectly affect subsequent well-being in ER through beliefs about the normative retirement age and negative stereotypes of older employees, which affect the timing of ER and subsequent well-being. Moreover, it should be noted that the financing mechanisms underlying retirement plans differ from one country to the next, as Hershey et al. (2013) suggested, and this should be taken into account when performing group comparisons.

The antecedent correlates-ER relationship was found to vary in terms of the participants' labor status, due to the fact that this implies different task characteristics and working conditions. Our results supported that the ES for the income-ER relationship was stronger for blue- collar workers, and the differences indicated the opposite direction for the subjective income measure, financial security. In the same vein, considering that blue-collar workers perform tasks with higher physical demands, stronger ESs would be expected for the poor physical health-ER relationship, and the results indicate this pattern. Finally, differential job features suggested that the job stress-ER relationship would differ between white- and blue-collar workers. Our results showed higher values for blue-collar workers, although the findings are inconclusive. This may be due, on the one hand, to the existence of a wide range of occupational conditions that have been categorized as white-collar or blue-collar, and diverse factors within these categories could affect the relationships between antecedent or subsequent correlates and ER. Recent studies showed that long-term exposure to low job complexity, i. e.: industrial production work has detrimental effects on cognitive functioning and regional gray matter volume, and it would have an impact on retirement decision making (Oltmanns et al., 2017). As Feldman (2013) stated, workers make appraisals about how unusual it is for someone their age to still be in the workforce, but these evaluations refer to a concrete occupational field.

Moreover, regarding sample comparisons, it should also be considered that people who are far away for retirement would view it as a reaction to their job features. On the contrary, people who are close to retirement “envision retirement primarily in terms of desirability/undesirability of the new role,” as Barnes-Farrell (2003, p. 173) stated. Hence, any comparison between white-collar and blue-collar workers should consider retirement timing. For instance, other factors could be affected, such as the social presentation of early retirees, in the sense that ER among blue-collar workers is socially more accepted than among white-collar workers (Schreurs et al., 2011). Moreover, these categories can be misleading; as there are highly skilled blue-collar workers (i.e., craftsmen) who tend to have more secure and stable working conditions and suffer from less alienation than routine blue- and white-collar workers (Williamson et al., 1992). So, ER decisions depend on a more complex pattern, and do not respond to this simple categorization into two types of employees.

Our meta-analyses advance the understanding of the impact of voluntariness on ER-antecedent correlates relationships, providing more evidence that the negative income-ER relationship will be stronger for forced early retirees, whereas financial security showed a stronger positive relationship for voluntary retirement. Regarding poor health measures, both physical and mental health proved to have a stronger influence on ER when the decision was voluntary rather than forced.

We also noted that participants' gender moderates the finances-ER relationships, showing a stronger impact both of objective and subjective measures on ER for males than for females. Concomitantly, previous studies suggested reduced financial preparation among women (Taylor and Geldhauser, 2007). Our results provide meta-analytical support to the statements which refer too many reasons—both psychosocial and economical—for gender to have an impact on ER. Men and women lead differentiated social roles that would lead the latter to a discontinuous work trajectory, where factors such as caregiving could affect their decision to retire. In turn, the impact of gender on subsequent retirement decision-making may be mediated by domestic responsibilities, the amount of expected pension at retirement, and the centrality of work in their lives. Moreover, the specific relationship between financial security and ER could be affected by the impact of discontinuous and shorter women's working careers. Perhaps these unclear results support the hypothesis that women tend to deny the information about their future financial hardship in retirement but, in fact, women's pensions will be lower than men's (Topa et al., 2017b).

A substantial body of research exists on the impact of chronological age on ER, and this research consistently shows that age plays a major role in understanding ER. Our results showed that finances, both objective and subjective, showed less impact for those aged below 55 years, whereas poor health measures showed the opposite pattern. These findings are in line with previous research on the relationships between age and a broad range of work-related variables (van der Heijden et al., 2009; Kooij et al., 2010, 2011; Mueller et al., 2013). In this case, mean age would be considered as a proxy variable that masks the existence of various individual perceptions and feelings about whether it is time to retire, along with different normative assumptions of what “early” retirement is.

## Limitations

As mentioned above, several limitations should be recognized in this study. One of the most relevant sources of difficulty was that, despite the increasing amount of studies on ER, only a limited number of empirical papers test each pair of relationships between variables. Perhaps this is due to that only one or two theoretical frameworks on ER have been offered as a base for primary studies. Moreover, a great amount of empirical evidence has been based on retirees' perceptions as a source of information, without including data from other sources, as supervisors, coworkers, or relatives. This procedure precludes the possibility of distinguishing personal perceptions from more objective ER features.

Secondly, we could only apply the moderator analysis to a limited amount of pairs of variables, due to lack of primary studies in the diverse categories. Although potential moderator factors were examined, it is clear that there are several unknown or not-measurable factors which contribute to ER and about which there are very little data available. As a consequence, our conclusions reached from these analyses have been limited and the moderating effects seemed to be a bit confusing. Again, we strongly recommend future empirical research on ER that deeply analyzes those underexplored relationships.

Despite these limitations, this is a comprehensive review of the most recent literature on the topic and we think that it helps the reader to gain an exhaustive overview of the main antecedent and subsequent correlates as well as of potential moderators of ER.

## Practical implications

Taken together, the results of our study show the many causes that characterize the antecedent and subsequent correlates of ER. Given that older workers tend to remain longer in the labor market than in previous decades—which represents a shift from “pro-retirement” to “pro-work” for older workers in the context of retirement (Wang and Shultz, 2010)—, it is a priority to design working conditions and organizational contexts aimed at mitigating the declines in the adjustment between the person and the environment over time for workers in their mid and late career, as negative attitudes toward older workers (Fasbender and Wang, 2017; Beléndez et al., 2018). As recent studies suggested, respect from leaders (Wöhrmann et al., 2017) and organizational climate would have an impact on retirement decision-making (Zacher and Yang, 2016).

One of the more important practical implications is the recommendation to avoid involuntary ER, given the negative effects it has on variables such as health, financial status, and adjustment to retirement. Like other forms of involuntary employment exit, involuntary ER may impact negatively on health through increased stress or the adoption of poor health-related behavior (Bartley, 1994), and it can often lead to material deprivation through the reduction of income. However, it can also lead to other forms of deprivation. Employment provides a number of non-economic latent functions (Jahoda, 1982), such as a daily structure and regular activity, a sense of self-efficacy, or identity and social status, which are lost when an individual is forced out of work. Human Resource Management practices designed for older workers (i.e., ER programs, among others) are perceived as a sign that they belong to a devalued social group (Hennekam and Herrbach, 2015; Kmicinska et al., 2016; Derous and Decoster, 2017). As a result, organizations should avoid forcing workers who show resistance to ER programs to retire, because the adverse effects on the individuals, their families, and health and social protection systems are disproportionate in comparison with the economic gain of this measure.

These issues, in combination with extrinsic or intrinsic motives, have relevant practical implications in the decisions to prolong working life, for example, through modalities of bridge employment. One study (Dingemans and Henkens, 2014) shows that participation in bridge employment for financial motives was associated with decreases in life satisfaction compared with postretirement-working based on intrinsic motives. In addition, compared with voluntary retirement, involuntary retirement was detrimental to life satisfaction, but participation in a bridge job was found to mitigate this negative shock. As Feldman (2013) argues, declining in the adjustment between older workers and their environment would not, in and of itself, be the only predictor of ER. A subjective comparison with alternative states or roles can be determinant. So, if older workers with poor adjustment to their environment perceive that fit with bridge employment role or full withdrawal role would be even worse, they may not choose to retire voluntarily. In sum, declines in different types of adjustment between older workers and their environment would lead to different ways of work-exit strategies (e.g., ER, retirement at mandatory age, prolonging working life via career or non-career bridge jobs—immediate or delayed, steady or intermittent, self-employed or other employed, same or different employers, as so on; see Beehr and Bennett, 2015). These different exit strategies depend upon the various opportunities open to older workers in their mid and late careers and the context constraints in which they operate (Henry et al., 2017).

One last practical implication is to note that ER is not necessarily the point of no return in working life, but a stage of transition before re-entry into working life at an older age, a trend endorsed by recent data (Cahill et al., 2013). If people accepted ER while still young (for example, before their 60s), going back to work under certain conditions is very feasible and beneficial to the individuals, the organizations, and the social systems and pension systems in countries with a more aged population.

## Perspectives about early retirement and future research

Although ER was a characteristic of the last two decades of the twentieth century, it is obvious that in the past few years, the tendency has reversed, and a marked preference by older workers and organizations toward maintaining the labor force and prolonging work life is detected. The data indicate that although the labor market participation of older workers is considerably lower than the labor market participation of prime-age workers, this gap declined considerably from 1995 until 2014, and the trend toward the ER options, promoted by governments in order to avoid high unemployment rates mainly among young people, has halted and even gone into reverse, as has been observed in Europe and the United States and in other regions of the world (Saba and Guerin, 2005; Cahill et al., 2006; Kantarci and van Soest, 2008; Quinn, 2010). One of the factors that may have contributed to the decline in early labor market withdrawal is the rise of non-standard forms of employment among older workers, which can be linked to the increase of bridge employment modalities (Feldman, 1994; Wang et al., 2008).

In the near future, ER will continue to be a formula used by older workers and organizations to deal with known needs and situations. However, we think that its use will be much more selective and restricted, because the need to cope with population aging, organizations' need to retain highly qualified older workers, maintaining the pension systems, and the promotion of active aging policies and practices will further the use of formulas designed to encourage *late careers* (e.g., Engelhardt, 2012), *post-retirement employment* (e.g., Pleau and Shauman, 2013), *gradual retirement* (e.g., Kantarci and van Soest, 2008), *working longer* (e.g., Munnell et al., 2008), *labor force re-entry* (e.g., Cahill et al., 2011), *employment of early retirees* (Mulders et al., 2014), and *bridge employment* (Wang et al., 2008). As a consequence, it is plausible to anticipate that knowledge acquired about the antecedent and subsequent correlates of ER will serve to design more efficacious formulas and to optimize their use so they will benefit the people and the organizations, as well as society as a whole. As Fisher et al. (2016) have noted, there are both benefits and drawbacks related to ER, as well as to on-time and later retirement, and there are potential tradeoffs and losses associated with retirement at various time points. The goal of this meta-analytical study was to help identify and better understand these positive and negative factors. We hope to have made this contribution to the literature.

## Author contributions

All authors listed have made a substantial, direct, and intellectual contribution to the work, and approved it for publication.

### Conflict of interest statement

The authors declare that the research was conducted in the absence of any commercial or financial relationships that could be construed as a potential conflict of interest.
